# New York State’s Paid Family Leave Program is Associated with More Equitable and Increased Use of Paid Leave Following Childbirth

**DOI:** 10.1007/s10995-022-03510-6

**Published:** 2023-01-07

**Authors:** Trang Nguyen, Barbara A. Dennison, Anne Radigan, Eileen FitzPatrick, Wei Zhang, Butho Ncube

**Affiliations:** 1 Office of Public Health, New York State Department of Health, Albany, NY USA; 2 Department of Epidemiology and Biostatistics, University at Albany School of Public Health, Rensselaer, NY USA; 3 Department of Health Policy, Management and Behavior, University at Albany School of Public Health, Rensselaer, NY USA; 4 Baby-Friendly USA, Albany, NY USA

**Keywords:** Parental leave, Maternity leave, Paid leave, Health policy, Women's health, Maternal and child health, Disparities, Health equity

## Abstract

**Objectives:**

This study aimed to assess changes in paid maternity leave before and after New York’s (NY) Paid Family Leave (PFL) law went into effect (1/1/2018) and changes in disparities by maternal characteristics.

**Methods:**

We used specific data collected on maternity leaves by women who gave birth in 2016–2018 in NY State (outside NY City) participating in the Pregnancy Risk Assessment Monitoring System survey. Multiple logistic regressions were conducted to evaluate the effect of the PFL law on prevalence of paid leave taken by women after childbirth.

**Results:**

After NY’s PFL law went into effect, there was a 26% relative increase in women taking paid leave after childbirth. Use of paid leave after childbirth increased among all racial and ethnic groups. The increases were greater among Black non-Hispanic or other race non-Hispanic women, compared to white non-Hispanic women, suggesting that NY’s law was associated with more equitable use of paid leave following childbirth.

**Conclusions for Practice:**

Wider implementation and greater utilization of paid maternity leave policies would promote health equity and help reduce racial/ethnic disparities in maternal and child health outcomes.

## Significance Statement

Despite strong evidence that paid leave after childbirth is associated with improved maternal and infant health, the US has no federal program for paid leave after childbirth. When NY’s PFL program became effective, it was the fifth such state PFL program. Uptake of PFL benefits in other states has been reported as slow and uneven, due, in part, to low awareness of the state PFL program, especially among people of color, workers with lower educational attainment or those who earn lower wages. This study examines the early impact of NY’s PFL program on leave-taking by women after childbirth.

## Introduction

Paid family and medical leave are increasingly gaining support as an important strategy to improve the health of children and their families. The number of state and municipalities implementing Paid Family Leave (PFL) programs has increased dramatically in the United States (U.S.) (A Better Balance, 2021). At the federal level, President Biden’s administration included PFL in the 2021 federal American Families Plan proposal to help support children, families and the U.S. economy (The White House, 2021).

Multiple studies find that paid leave after childbirth is associated with better maternal and infant health, and that women who take a longer duration of leave breastfeed longer, are more likely to meet their breastfeeding goals, and have lower rates of maternal or infant rehospitalization and use of maternal mental health care (Andres et al., 2016; Burtle & Bezruchka, 2016; Jou et al., 2018). Despite these benefits, the U.S., Oman and Papua New Guinea are the only countries worldwide that do not have a national program to provide paid leave to women after childbirth (International Labour Organization, 2014).

In the U.S., the federal Family and Medical Leave Act (FMLA) provides qualified workers with up to 12 weeks of job-protected, unpaid leave for specified family or medical conditions (U.S. Department of Labor, n.d.). But only about 60% of U.S. workers are eligible to take leave under FMLA (Boesch, 2019).

Access to PFL is limited in the U.S., with only 15% of workers having access to employer-sponsored PFL, which is available primarily to high-paying, professional employees, working at larger companies (Bartel et al., 2019; The Economics Daily, 2019). Some employees/workers also have access to other types of employer-sponsored paid leave, such as paid time off, vacation time, sick leave, or personal days.

Workers in five U.S. states—California (CA), Hawaii (HI), New Jersey (NJ), New York (NY), and Rhode Island (RI)—and the U.S. territory of Puerto Rico—may have access to limited paid leave before and after childbirth as a part of their state’s/territory’s Temporary Disability Insurance (TDI) programs (Eligibility Team, 2017). TDI benefits vary by program; for example, eligible workers in NY and CA may take 2–4 weeks of TDI leave before their due date and 4–6 weeks leave after their delivery date. The benefit is paid at a maximum of 50% of their average wages, but in NY the benefit is capped at $170 per week, while in CA, the TDI and PFL benefits are equal (Upcounsel, n.d.).

Before 2018, four U.S. states had state family and medical leave laws (effective dates): CA (2004); NJ (2009); RI (2014); and Washington (WA) (2015) (National Conference of State Legislatures, 2022). The provisions of the state programs vary by conditions covered (care of newborn, adopted or fostered child; care of family member with serious illness; military exigencies, etc.), duration of leave and benefit level (National Conference of State Legislatures, 2022; Bartel et al., 2016). At that time, only RI’s PFL program provides job protection.

To date, CA’s PFL program is the best studied, with limited reports from NJ and RI. Low awareness of state PFL programs can hinder utilization. In CA, studies indicate that uptake of PFL benefits was slow and uneven. In both CA and NJ, there was less awareness of their state’s PFL program among people of color, workers with lower educational attainment and those who earn lower wages (Appelbaum & Milkman, 2011; Houser & White, 2012). The PFL programs in CA and NJ lack job protection, which may also contribute to low uptake.

On January 1, 2018, NY’s PFL program took effect, making NY the fifth state to enact a state PFL law, that covers leave to bond with a newly born, adopted or fostered child; care for a family member with a serious health condition; or assist loved ones when a spouse, domestic partner, child or parent is deployed abroad on active military service (New York State Paid Family Leave, 2018). The NY PFL law aims to cover most employees and reduce barriers for employees to take leave by providing stronger protections than other states, including continuing health insurance, guaranteeing the same or comparable job after the leave ends, and protecting against discrimination or retaliation for requesting or taking PFL (New York State Paid Family Leave, 2018). In 2018, the NY PFL benefit provided up to 8 weeks leave, paid at 50% wage replacement, capped at the NY State Average Weekly Wage ($1305.92; maximum benefit was up to $652.96 per week) (New York State Paid Family Leave, 2018). In 2018, NY PFL provided more weeks of paid leave than the PFL programs in NJ, RI or WA; in 2019, the same number of weeks as CA; and in 2021, more weeks than CA (Rossin-Slater & Uniat, 2019). Between 2018 and 2021, the NY PFL benefit increased up to 12 weeks leave, paid at 67% wage replacement (2021).

To better understand the early impact of the NY’s PFL law, our study aimed to compare maternal use of paid leave following childbirth (i.e., maternity leave or newborn bonding leave) before and after NY’s PFL law took effect. We also examined whether changes in the use of paid leave differed across socio-economic and demographic characteristics, assessed changes in the total leave taken after childbirth, and changes in factors affecting working individuals’ decisions about taking leave after childbirth.

## Methods

### Data Sources

We used three years of data (2016–2018) from the NY State Pregnancy Risk Assessment Monitoring System (PRAMS), a population-based mail/telephone survey of women who have recently given birth to a live born infant in NY, outside New York City (NYC). Women are surveyed 2–4 months postpartum, to obtain information about their experiences and behaviors before, during and after pregnancy (Centers for Disease Control and Prevention, n.d.). Starting in 2016, we added specific questions to the NY State PRAMS survey to ask about employment during pregnancy, use of paid leave, unpaid leave and/or no leave taken after the child’s birth, duration of total leave after childbirth, and factors that might impact their decisions about taking leave from work. The duration of leave taken was not ascertained separately by whether it was paid or not. The NY State PRAMS dataset also included other information such as maternal age, race/ethnicity, educational attainment, marital status, smoking history, participation in the Special Supplemental Nutrition Program for Women, Infants and Children (WIC), health insurance during pregnancy, and delivery method.

### Study Design and Population

**Fig. 1:**
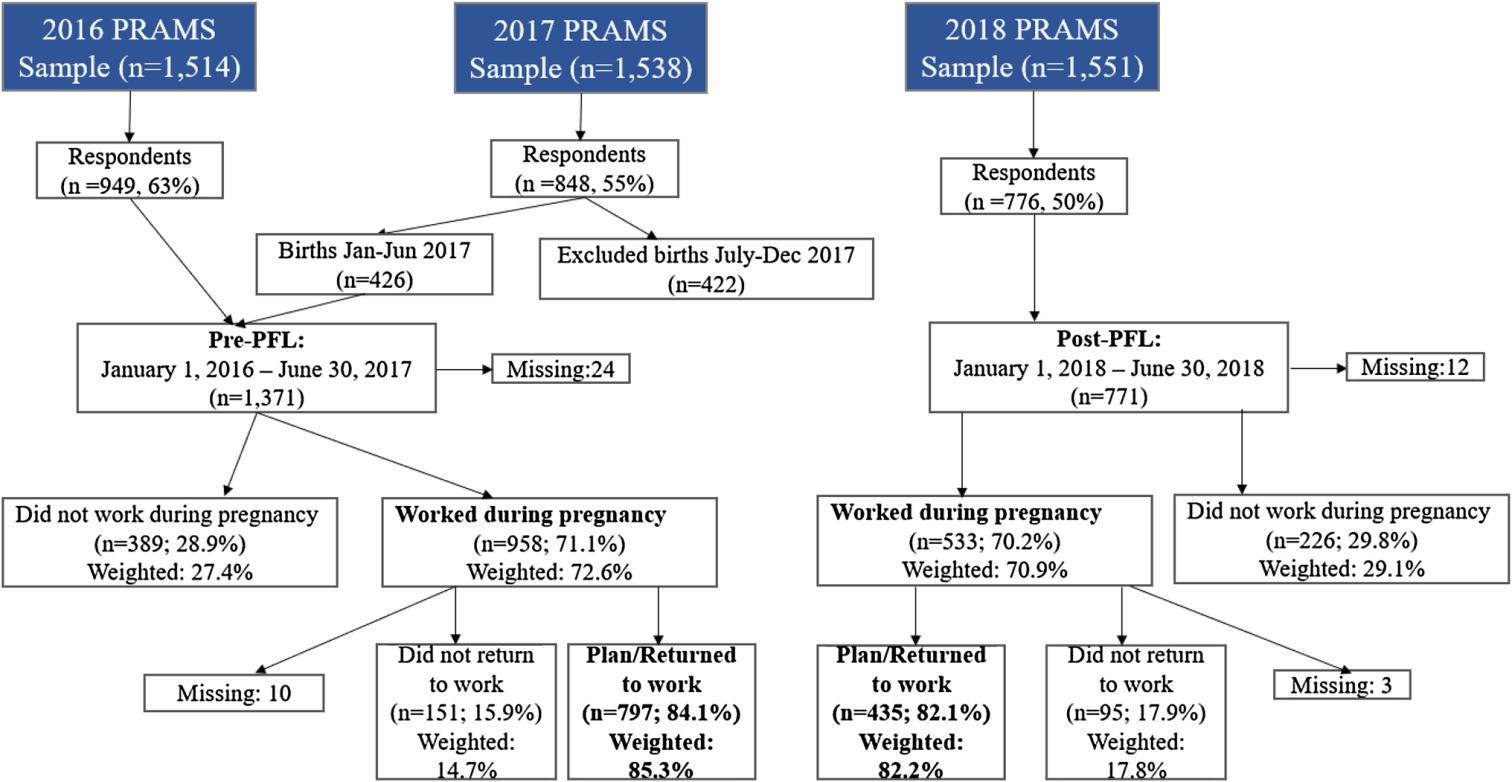
New York State Pregnancy Risk Assessment Monitoring System (PRAMS) samples, 2016–2018, and study populations. Weighted percentage of women who worked during pregnancy, Pre-PFL vs. Post-PFL: 72.6% vs. 70.9%; Chi-square P-value = 0.54. Weighted percentage of women who returned or planned to return to work, Pre-PFL vs. Post-PFL: 85.3% vs. 82.2%; Chi-square P-value = 0.24

We used a pre-post study design to assess differences, between the pre-PFL and post-PFL time periods, in the use of paid leave and factors influencing women’s decisions to take leave after childbirth. The study population did not include women residing in NYC because their surveys were collected separately by the NYC Department of Health and Mental Hygiene and were not asked these paid leave-related questions. We included infants born between January 1, 2016, and June 30, 2017, in the pre-PFL study populations, and infants born between January 1, 2018, through December 31, 2018, in the post-PFL study population (Fig. 1). Parents may file a NY PFL claim up to 1 year after the birth of a newborn, thus parents with infants born in 2017. may file claims in 2018 for newborn bonding. We excluded births between July 1 and December 31, 2017. and considered this a transition period. Women who reported not working during this pregnancy were excluded, as they would not be eligible to take NY PFL. Thus, the final dataset included women who reported working during this pregnancy who planned to or had already returned to work.

### Outcome Variables

Our primary outcome measure was paid leave taken after childbirth, or paid maternity leave, defined as taking any type of paid leave from work after childbirth. We classified women who reported taking both paid leave and unpaid leave as taking paid leave; those who reported taking only unpaid leave (but no paid leave) as taking only unpaid leave; and those who reported taking only no leave as such. Our secondary outcomes are changes, pre-PFL compared to post-PFL, in the frequency of potential barriers that may have impacted a woman’s decisions about taking leave after childbirth, such as not having paid leave, not having enough leave saved, not being able to financially afford it, having too much work to do, being afraid of losing one’s job, or job not having a flexible schedule.

### Independent Variables

Several socio-demographic characteristics have been associated with women’s access to and use of leave after childbirth. Specifically, women who are older, married, highly educated, have private health insurance, have higher incomes, or are non-Hispanic white are more likely to have access to paid leave and take longer maternity leaves (Bartel et al., 2019; The Economics Daily, 2019; Horowitz et al., 2017; Zagorsky, 2017). We included these variables (maternal age, race/ethnicity, educational attainment, marital status, insurance, WIC participation, birth born indication and delivery method) as covariates in the analyses.

### Statistical Analyses

We compared the weighted distributions of maternal characteristics, delivery method and total leave taken after childbirth between the pre-PFL and post-PFL populations using Chi-square and T-tests. We calculated the weighted percentage of women taking paid leave (prevalence) and the relative percentage changes for the entire pre-PFL and post-PFL populations, and for subgroups (based on maternal characteristics). To assess changes in taking paid leave after childbirth between the pre-PFL and post-PFL populations, we computed the crude and adjusted prevalence ratios (PR)s, with 95% confidence intervals (CI) for total and subgroups. We generated the adjusted PRs using weighted logistic regression, and a special survey-specific analytic software program (in SUDAAN), to adjust for multiple maternal characteristics described above. Finally, we examined the changes in paid leave taken, measured by the pre-PFL and post-PFL adjusted PRs, using weighted logistic regression adjusted for other maternal characteristics. We conducted data linking, processing and cleaning using SAS software.

### Limitations

An inherent limitation of using self-reported data is the possibility of recall or social desirability bias. The PRAMS survey is conducted soon (i.e., two to four months) after childbirth, reducing the risk of recall bias. Questions about employment and intention to return to work are not as sensitive to social desirability bias. The sample sizes, especially for the post-PFL law period were smaller, but there was sufficient statistical power for the major pre-to-post comparisons. The PRAMS survey question about the use of paid leave following childbirth is a single question that does not inquire separately about different types of paid leave (e.g., sick time, vacation leave, NY PFL, or employer-sponsored maternity leave). However, we expect that this would lead to non-differential misclassification of outcomes, which would tend to reduce any associations (Godolphin et al., 2020). Finally, the study sample represents the NY State population outside New York City; therefore, the results may not be generalizable to populations from New York City or other states.

This research was approved by the Institutional Review Board of the New York State Department of Health.

## Results

The PRAMS samples for 2016–2018, the response rate, and the final pre-PFL (2016–2017; N = 1371) and post-PFL (2018; N = 771) study populations for data analysis are shown in Fig. 1. The weighted percentages of women who worked during pregnancy or who planned/returned to work after childbirth were similar pre- and post-PFL.

The pre-PFL and post-PFL study populations, of women who worked during pregnancy, did not differ in the weighted distributions of maternal age, educational attainment, marital status, Medicaid insurance, WIC participation during pregnancy, first birth status, or delivery method (Table 1). The racial/ethnic distribution, however, differed slightly with the post-PFL sample including a higher percentage of white non-Hispanic and Black non-Hispanic respondents and a lower percentage of Hispanic respondents compared to the pre-PFL sample (P-value = 0.023).

**Table 1:** Characteristics of women who worked during pregnancy (Pre-PFL vs. Post PFL), New York State Pregnancy Risk Assessment Monitoring System, 2016–2018 (N = 1491)

Maternal characteristics	Pre-PFL^a^ n = 958 (%)^c^	Post-PFL^b^ n = 533 (%)^c^	P-value^d^
Race/ethnicity			0.023
White non-Hispanic	679 (70.6)	376 (74.6)	
Black non-Hispanic	82 (6.5)	73 (10.3)	
Other non-Hispanic	78 (8.3)	31 (5.0)	
Hispanic	119 (14.6)	53 (10.2)	
Age (years)			0.325
≤ 24	151 (17.5)	83 (21.4)	
25–34	546 (57.9)	304 (53.6)	
35 +	261 (24.6)	146 (25.0)	
Educational attainment			0.875
High school graduate or less	202 (25.2)	112 (24.2)	
Some college	247 (25.9)	147 (26.5)	
Bachelor’s degree	233 (22.6)	123 (20.8)	
Master’s or Doctoral degree	272 (26.4)	147 (28.4)	
Marital status			0.453
Married	634 (60.1)	333 (62.8)	
Not married	324 (39.9)	200 (37.2)	
Insurance			0.574
Not medicaid	680 (68.8)	392 (70.7)	
Medicaid	278 (31.2)	141 (29.3)	
Women on WIC^e^ during pregnancy			0.843
Participated	242 (28.5)	139 (27.8)	
Did not participate	702 (71.5)	383 (72.2)	
Delivery type			0.576
Vaginal	533 (64.9)	307 (66.8)	
Cesarean section	424 (35.1)	225 (33.2)	
First birth status			0.197
Not first birth	501 (59.2)	280 (54.7)	
First birth	452 (40.8)	253 (45.3)	

^a^Pre-PFL: Women giving birth before NY’s paid family leave (PFL) Law was effective (01/01/2016–06/30/2017)

^b^Post-PFL: Women giving birth after NY’s PFL Law was effective (01/01/2018–12/31/2018)

^c^Weighted percent

^d^Chi-square test

^e^WIC = Special supplemental nutrition program for women, infants, and children

### Taking Paid Leave

After the NY PFL law became effective, the relative percentage increase in the use of paid leave after childbirth was 26.2% (from 55.8 to 70.4%) accompanied by a 39% relative percentage decrease in use of unpaid leave alone (from 40.1 to 24.5%) (Table 2). There was no pre-post difference in the percentage of women reporting “no leave” (4.1% versus 5.1%) There was no change in the mean duration of total leave taken: 12.3 weeks for pre-PFL vs. 12.1 weeks for post-PFL (P-value = 0.62); and no changes were observed by maternal demographic variable (Table 2). Pre-PFL, there were no significant associations between the maternal characteristics listed and the use of paid maternity leave (i.e., the adjusted PRs did not differ across stratified maternal subgroups). Post-PFL, however, use of paid maternity leave was significantly higher among women of color; the adjusted PRs for Black non-Hispanic women and Other non-Hispanic women were 1.39 and 1.40, respectively, compared to white non-Hispanic women, after adjusting for other independent variables. Among all subgroups, there was an increase in the percentage of women taking paid leave after childbirth, post-PFL vs. pre-PFL. The relative percent changes, however, differed by maternal characteristics. The largest relative percentage increase (post-PFL vs. pre-PFL) was observed among Black non-Hispanic women (73%), compared to a 17% relative percentage increase among white non-Hispanic women.

**Table 2:** Percentages and prevalence ratios (PR) of women taking paid leave after childbirth, New York State Pregnancy Risk Assessment Monitoring System, 2016–2018

Maternal characteristic	Paid leave Pre-PFL^a^			Paid leave Post-PFL^b^			Relative % change in paid leave (Post-PFL—Pre-PFL)
	Leave duration (weeks)	Paid leave (%)^c^	Adjusted PR^d^ (95% CI)	Leave duration (weeks)	Paid leave (%)^c^	Adjusted PR^d^ (95% CI)	
Total	12.3	55.8		12.1	70.4		26.2
Race/ethnicity							
White Non-Hispanic*	12.3	57.2	1	12.2	67.1	1	17.3
Black Non-Hispanic	10.6	52.3	1.05 (0.78–1.42)	12.1	90.5	**1.39 (1.20–1.62)**	73
Other Non-Hispanic	14.3	64.8	1.18 (0.94–1.50)	12.8	89.9	**1.40 (1.17–1.68)**	38.7
Hispanic	12	44.6	0.87 (0.63–1.19)	10.7	66.3	1.09 (0.83–1.42)	48.7
Age (years)							
< 24	9.2	44.2	0.89 (0.66–1.20)	8.6	56.1	0.85 (0.62–1.17)	26.9
25–35*	12.1	58.6	1	12.1	74.9	1	27.8
35 +	14.4	55.8	0.94 (0.79–1.13)	14.3	70	0.92 (0.77–1.10)	25.4
Educational attainment							
High school graduate/GED or less	9.8	44.5	0.78 (0.57–1.07)	9	58.1	***0.72 (0.53–0.98)***	30.6
Some College	10.5	52.1	0.83 (0.66–1.03)	11.1	66.7	***0.74 (0.59–0.94)***	28
Bachelor's Degree*	12.5	64.7	1	14.2	84.2	1	30.1
Masters or Doctoral Degree	15.4	61.1	0.92 (0.76–1.12)	13.2	71.9	0.89 (0.76–1.04)	17.7
Marital status							
Married*	12.7	58.3	1	12.3	68.9	1	18.2
Not married	11.6	51.9	1.02 (0.84–1.24)	11.6	73.7	1.18 (0.98–1.41)	42
Women on WIC^e^ during pregnancy							
Yes*	9.9	46.3	1	9.2	62.2	1	34.3
No	13.1	59.6	1.12 (0.86–1.44)	13	73.1	1.12 (0.83–1.51)	22.7
Method of delivery							
Vaginal	12	54	1.07 (0.91–1.26)	12	70.3	0.99 (0.85–1.16)	30.2
C-section*	12.6	59.7	1	12.1	70.6	1	18.3
First birth status							
First birth	12.3	58	1.00 (0.85–1.17)	12.2	74.5	0.93 (0.79–1.09)	28.4
Not first birth*	12.2	55	1	12	67.3	1	22.4

*Reference group

^a^Pre-PFL: Women giving birth before NY’s paid family leave (PFL) Law was effective (01/01/2016–06/30/2017)

^b^Post-PFL: Women giving birth after NY’s PFL Law was effective (01/01/2018–12/31/2018)

^c^Weighted percent

^d^Adjusted prevalence ratios and 95% confidence intervals were generated using weighted logistic regression models

^e^Prevalence ratios are bolded if significantly different from 1 (confidence interval does not include 1). Prevalence ratios are bolded and italicized when the ratios are significantly lower than 1

**Fig. 2:**
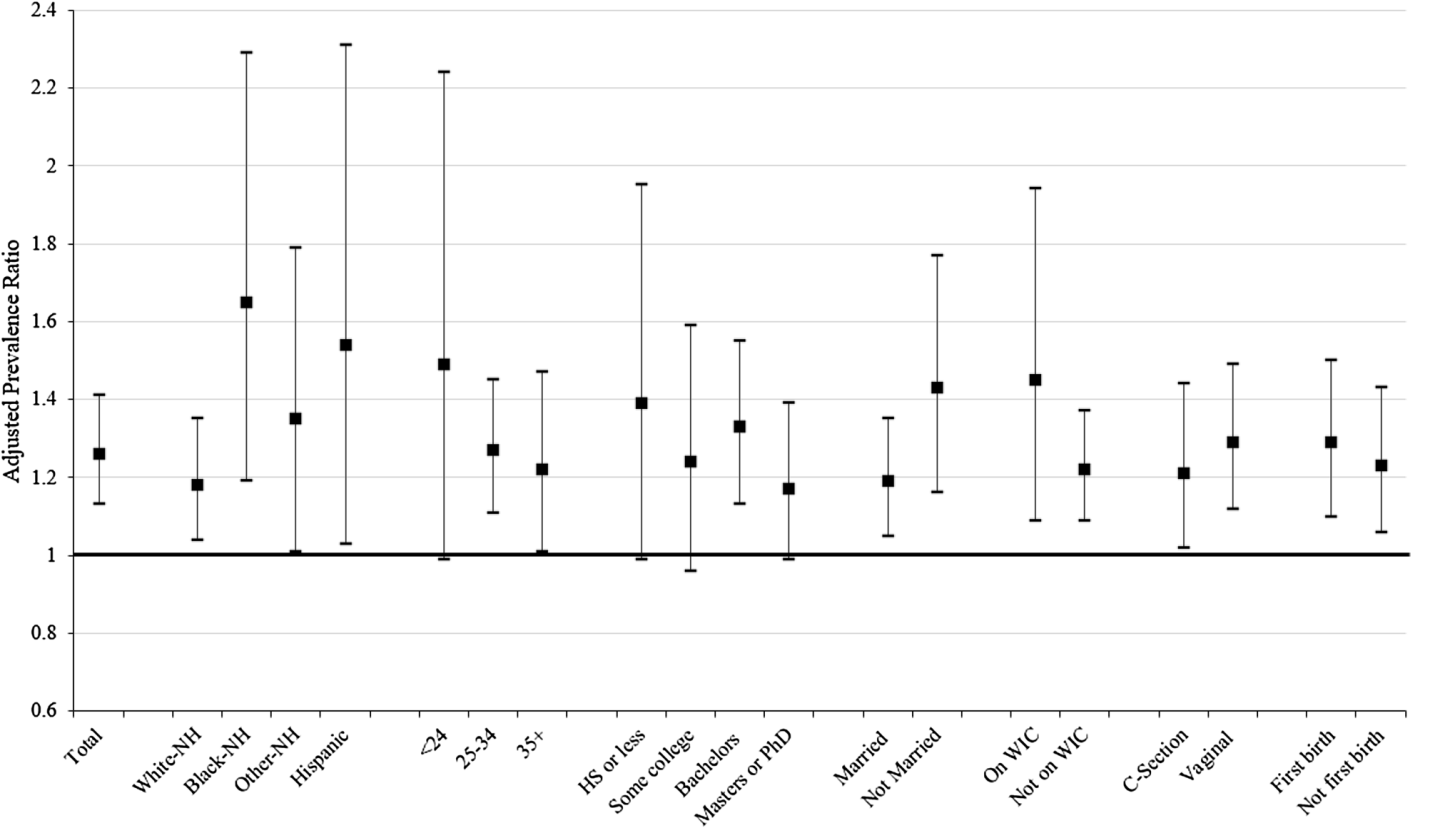
Adjusted prevalence ratios (Post-PFL to Pre-PFL) of women taking paid leave after childbirth, New York State Pregnancy Risk Assessment Monitoring System, 2016–2018. Adjusted prevalence ratios and 95% confidence intervals were generated using weighted logistic regression models

The adjusted PRs in Fig. 2 illustrate increases in the use of paid leave among the post-PFL population, compared to the pre-PFL population, for all women and across subgroups. Overall, 26% more women took paid leave after childbirth post-PFL compared to pre-PFL (PR = 1.26). Significant increases in use of paid maternity leave (post-PFL compared to pre-PFL) were seen among white non-Hispanic, (PR = 1.18), Black non-Hispanic (PR = 1.65), Other non-Hispanic (PR = 1.35), and Hispanic (PR = 1.54) women; women with a bachelor degree (PR = 1.33); women aged 25–34 years (PR = 1.27) or aged 35 + years (PR = 1.22); women who delivered vaginally (PR = 1.29), or by C-section (PR = 1.21); women who were married or not married (PR = 1.19 and 1.43, respectively); women who did and did not participate in the WIC program during pregnancy (PR = 1.45; and 1.22, respectively); and women delivered first child or not first child (PR = 1.29; and 1.23, respectively). While the use of paid leave increased among the other subgroups, the confidence intervals were wide, due to small sample sizes, and the differences were not statistically significant.

### Factors Affecting Decisions to Take Leave After Childbirth

**Fig. 3:**
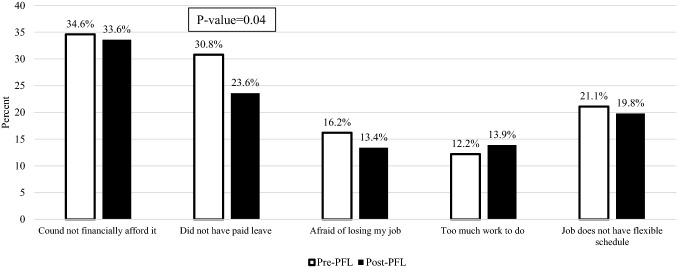
Factors affecting women’s decision to take leave after childbirth (Pre-PFL vs. Post-PFL), New York State Pregnancy Risk Assessment Monitoring System, 2016–2018. Pre-PFL: women giving birth before NY’s paid family leave (PFL) Law was effective (01/01/2016–06/30/2017). Post-PFL: Women giving birth after NY’s PFL Law was effective (01/01/2018–12/31/2018). Weighted percent, Chi-square test

Several factors affect an individual’s decision to take maternity leave, shown in Fig. 3. The percentage of women who reported each factor did not change significantly, pre-PFL vs. post- PFL, except concerns about “not having paid leave” which decreased significantly (30.8% vs. 23.6%, P-value = 0.04). The percentage of women reporting they were “afraid of losing [one’s] job” decreased from 16.2% pre-PFL to 13.4% post-PFL. However, this reduction was not statistically significant.

## Discussion

Paid maternity leave after childbirth has been associated with improved maternal and infant health (Bullinger, 2019; Doran et al., 2020; Jou et al, 2018; Lee et al., 2020). Our study shows that during the first year that the NY PFL law was in effect, there was a significant change in the type of leave used by women following childbirth, with more working women taking paid leave, while fewer used only unpaid leave. After adjusting for major demographic factors, we saw a 26% relative increase in use of paid leave. Prior to NY PFL being in effect, 56% of working women reported taking some type of paid leave after childbirth, which is consistent with two national studies: 48% (Zagorsky, 2017) and 58% (Goodman et al., 2020b). After NY PFL took effect, the percentage of working women reporting taking paid leave after childbirth increased significantly to 70%.

While we cannot say that the NY PFL law directly caused the increase in the percentage of women taking paid leave after childbirth, we do find a temporal association. Our finding that the percentage of women reporting two barriers that affect a woman’s decision to take leave were directly impacted by the NY PFL law (i.e., “not having paid leave” and being “afraid of losing [one’s] job”) decreased after the law took effect, provide additional temporal support for an association between implementation of the NY PFL law and increased use of paid leave. We do not know whether the provision of job protection, protection against discrimination and/or retaliation for requesting or taking PFL influenced a woman’s decision to take maternity leave.

Previous literature finds disparities in access to and use of employer-provided paid maternity leave, with greater use by women who are white non-Hispanic, married, or highly educated (Zagorsky, 2017). In this study, before NY PFL was in effect, we did not observe statistically significant associations between race/ethnicity, marital status, or educational attainment and the use of paid leave, after adjustment for covariates. After the NY PFL law took effect, we found that use of paid leave increased among all demographic groups, with greater increases among Black non-Hispanic and other non-Hispanic women, compared to white non-Hispanic women, and more among single women and women with lower educational attainment or lower incomes. These findings are consistent with the greater increases in use of paid maternity leave among less advantaged groups reported after CA’s PFL law took effect (National Partnership for Women & Families, 2015).

Despite some employers having expressed concern that employees might not return to work after taking PFL, there were no pre-post differences in the percentage of working women planning or returning to work after childbirth. There have also been reports that employer sponsored PFL programs increase job retention, especially among women.

In CA, even 15 years after the PFL program began, Goodman et al (2020a) reported low awareness and use of the PFL program by low-wage workers, immigrants, Hispanics, workers with less than a high school education, and those employed in smaller firms. In NJ, three years after implementation of the state’s Family Leave Insurance program, 60% of NJ residents indicated that they were not aware of the program, with greater lack of awareness among people of color, those earning less than $50,000 per year, and young (18–29 years), older (65 + years), or non‐partnered adults (Houser & White, 2012).

Utilization of state PFL during the first operational year appears to be higher in NY than in CA, NJ, and RI for child bonding and for care of a family member. Specifically, in 2018, a higher percent of eligible workers in NY filed a NY PFL claim for child bonding (1.18%; 86,500/8.5 million) (New York State Paid Family Leave, 2018), than was reported in CA, NJ and RI during their first operational years: 0.75%, 0.48%, and 0.50%, respectively (National Partnership for Women & Families, 2015). These findings suggest that utilization of PFL for child bonding in NY was 1.6, 2.5, and 2.4 times higher than in CA, NJ, or RI, respectively, during their first operational years.

The total duration of leave taken after childbirth did not change after NY PFL became effective; mean duration of total leave (paid or unpaid) was 12 weeks pre-PFL and post-PFL. In contrast, after CA PFL became effective in 2004, studies reported that total maternity leave increased 2.4 weeks (Bartel et al., 2016) and 3.2 weeks (Rossin-Slater et al., 2013). One should note, however, that the pre-PFL period for the CA studies was 20 years ago (i.e., 2000–2003), when the average leave taken by women after childbirth was 7.8 weeks (Baum & Ruhm, 2014). One recent national study reported the average leave taken from work following the birth or adoption of a child was 11 weeks for women and 1 week for men (Horowitz et al., 2017). Another study from the San Francisco Bay area of CA, found that 89% of women giving birth in 2016–2017 took 12 weeks of leave (Goodman et al., 2020b). Women with higher household incomes took longer leaves after childbirth; the mean leave was 6 weeks, 10 weeks, and 12 weeks for those earning < $30,000, $30,000–$74,4999, and $75,000 + per year, respectively (Horowitz et al., 2017).

In NY, for leaves, such as childbirth, that qualify under both NY PFL and FMLA, employers can require both leaves to run concurrently (New York State Paid Family Leave, n.d.). Thus, employees covered under FMLA might not gain any additional leave time from the NY PFL law; but they would benefit financially from the leave being paid.

The finding of a decrease in use of unpaid leave, increase in use of paid leave, and no change in total leave duration, leads us to hypothesize that some women substituted NY PFL (which provided up to $652.96 per week) for unpaid leave, or for NYS TDI (which provided a maximum of $170 per week). Because paid leave benefits are typically a fraction of an employee’s usual wage/salary, families might not be able to afford to take additional time off. Workers who are eligible for FMLA often take less than the full amount, with 80% citing financial reasons, noting they are not being able to afford to take more unpaid leave (Glynn, 2012).

In the U.S., 12 weeks of leave after childbirth has become the norm among female employees, whether covered by FMLA or not (U.S. Department of Labor, n.d.; Baum & Ruhm, 2014). But outside the U.S., the norm is higher. More than 50% of countries (98 of 185) provide at least 14 weeks of paid time off when an infant is born, with 31% of these countries (57 of 185) also providing at least two-thirds of previous earnings, which are two of the minimum standards of the International Labour Organization for optimal maternal and infant health (Addati et al., 2014).

### Strengths

This study has many strengths, which outweigh the limitations. We utilized the nationally recognized PRAMS dataset, which employs questions developed, standardized and validated by the Centers for Disease Control and Prevention. The overall PRAMS’ response rates were between 50 and 65%, which is considered relatively high for a population survey. Combining these data with a second dataset, which includes objective data from the NY State birth certificate, expands the study variables and allows confirmation and triangulation of some measures. The PRAMS and birth certificate data were available before and after NY PFL law took effect, used the same methodology, and included the same questions. The pre-PFL and post-PFL study populations had comparable demographics, except for slight differences in race/ethnicity, and the percentages of women who worked during pregnancy and planned/returned to return to work after childbirth were similar.

## Conclusions

Our study is the first to report observed changes, after NY’s PFL law took effect, in the type (paid or unpaid) and duration of leave taken by women after childbirth. The increase in the percentage of women taking paid leave and decrease in use of unpaid leave following combined with no change in total length of leave taken after childbirth, suggests that women are substituting NY PFL for unpaid leave and/or leave paid at a lower percentage of usual wages (such as NY TDI). The percentage of women using paid leave increased across all demographic groups, with greater increases by women of color, suggesting that the NY’s PFL law was associated with more equitable use of paid leave following childbirth. These findings support wider adoption of PFL laws in other states or the federal level. Strong PFL policies that include job protection, broad eligibility and widespread awareness are necessary to further increase use of paid leave for childbirth. Increased access and use of PFL programs is a promising public health strategy to address some of the social determinants of health and to reduce racial/ethnic disparities in maternal and child health (Burtle & Bezruchka, 2016).

## Data Availability

Not applicable.
